# Taste of Tradition: Perceptual Quality Aspects and Future Prospects of Three Swedish Classic Cheeses

**DOI:** 10.1002/fsn3.70913

**Published:** 2025-09-04

**Authors:** Julie Hirtz, Kalyani Douraquia, Madeleine Jönsson, Karin Wendin

**Affiliations:** ^1^ Department of Food and Meal Science Kristianstad University Kristianstad Sweden; ^2^ Department of Food Science and Technology Institute Agro Dijon Dijon France; ^3^ Department of Food Science and Technology University of Reunion Island's Engineering School Saint Pierre France; ^4^ Department of Food Science University of Copenhagen Frederiksberg C Denmark

**Keywords:** consumer liking, future food, hard cheese, sensory profile, traditional food

## Abstract

Traditional cheese production represents an important aspect of gastronomic heritage, blending cultural identity with sensory characteristics. This study investigates the sensory characteristics, consumer preferences, and physicochemical properties of three traditional Swedish hard cheeses—Grevé, Herrgård, and Präst—matured for 12 and 18 months. Using Quantitative Descriptive Analysis (QDA), instrumental color and texture profiling, and a hedonic consumer study, the research explores how cheese type and maturation influence sensory perception and liking. Präst18 cheeses, characterized by higher fat content and crumbliness, were generally preferred, while Grevé12 received the lowest liking scores. Texture and taste were the primary drivers of consumer acceptance, whereas appearance had limited influence. Instrumental and sensory data revealed significant correlations between protein/fat content and textural attributes such as hardness and crumbliness. The study also highlights the role of familiarity in consumer preference, with Swedish participants showing greater liking and recognition of the cheeses. Despite limited international familiarity, all cheeses were positively received, suggesting potential for broader market appeal. The findings underscore the importance of preserving traditional cheese characteristics while considering future innovations. Understanding sensory drivers and consumer expectations can support the development of sustainable, culturally rooted food products. This research contributes to the discourse on traditional food systems, emphasizing their relevance in contemporary food culture and their potential role in promoting sustainability and regional identity.

## Graphical abstract

This study assessed the sensory quality and consumer acceptance of three traditional Swedish cheeses to inform the development of new local products that emphasize tradition, familiarity, and sustainability, thereby enhancing the understanding of the role of traditional foods in the future.

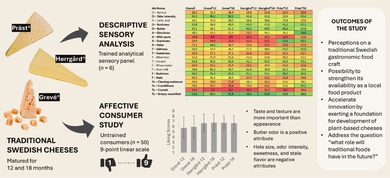

## 1 Introduction

Cheese making is an ancient method for preserving milk and is considered a traditional gastronomic craft found in almost all food cultures worldwide (Kindstedt 2012; Nilsson Blom and Weréen 2002). According to Fibri et al. (2022), traditional food is a blend of creations that cultivate local resources with flavorful legacies spanning many generations. It has further been defined through four key dimensions: product familiarity, preparation using traditional recipes, sensory properties, and geographical origin (Braghieri et al. 2014; Rocillo‐Aquino et al. 2021). Based on this, the current paper seeks deeper insight into current and future perspectives of traditional Swedish cheeses—Grevé, Herrgård, and Präst—by exploring sensory aspects, consumption frequency, and consumer likin. Quantitative descriptive analysis (QDA) is commonly used to develop sensory profiles in a standardized manner by defining sensory attributes and measuring their intensities on a scale (Lawless and Heymann 2010). This method can be further combined with a consumer study, such as a hedonic liking test using a 9‐point linear scale, to associate descriptive understanding with consumer acceptance (Lawless and Heymann 2010).

Only four out of ten cheeses consumed in Sweden today are produced within the country (Svenskmärkning AB 2024). Three of these are the hard cheeses Grevé, Herrgård, and Präst, which have registered trademarks owned by Svenska Ostklassiker AB and are licensed for manufacturing to companies like Allerum and Falbygdens ost. Grevé, developed between 1959 and 1964, was inspired by Swiss Emmentaler and Norwegian Jarlsberg and is characterized by a rich, sweet flavor and large, round holes (Svenska Ostklassiker AB 2020a). Similarly, Herrgård, also inspired by Swiss Emmentaler, is renowned for its mild flavor, versatility, and round holes. While it originated in 1786, its current recipe was refined a century later (Svenska Ostklassiker AB 2020b). Finally, Präst is one of the oldest Swedish cheeses, with a history dating back to the 16th century as a tithe paid by farmers to the priest (Swedish: präst). It is a creamy and flavorful cheese with small holes and a prevalence of crispy protein crystals (Nilsson Blom and Weréen 2002; Svenska Ostklassiker AB 2020c; Zheng et al. 2021).

While the ingredients are similar across different cheeses, variations in manufacturing and maturation processes render their distinctive characteristics. Principally, traditional animal‐based hard cheeses are formulated with four primary ingredients: milk, bacterial starter cultures, protein coagulation enzymes (rennets), and salt. Big round holes, as in Grevé and Herrgård, are formed when the curd is pressed under the whey in the absence of air. For Präst, the many small holes are formed in the presence of air when the whey is drained from the curd (Nilsson Blom and Weréen 2002; Zheng et al. 2021). Maturation (ripening) of hard cheeses can enhance the formation of specks and spots (macroparticles) as well as microcrystals (microparticles), which are defined as crystalline tyrosine, complex structures, and calcium phosphate crystals, respectively (D'Incecco et al. 2016). Lipolysis and proteolysis are crucial to the development of cheese flavor, and the ratio between protein and fat can impact the textural and organoleptic properties of cheese (Zheng et al. 2021). Generally, low fat content is associated with increased incidence of springiness, firmness (hardness), waxiness, and chewiness, as well as decreased cohesiveness, stickiness, smoothness, and meltability (Foegeding and Drake 2007).

This paper aims to explore and discuss perceptual quality aspects and prospects of traditional Swedish cheeses by investigating the consumption frequency, consumer acceptability, and sensory characteristics of three classic varieties—Grevé, Herrgård, and Präst—matured for 12 and 18 months.

## 2 Materials and Methods

### 2.1 Cheeses

This study examined three classical Swedish cheeses of the Allerum brand, matured for 12 and 18 months. This resulted in a total of six products: Grevé12, Grevé18, Herrgård12, Herrgård18, Präst12, and Präst18. According to the manufacturer, the chemical composition of the cheeses varied slightly, as indicated by the nutritional value tables on their packaging. All Grevé and Herrgård cheeses contained 27% protein and 28% fat, of which 18% consisted of saturated fat. The corresponding composition of both Präst varieties was 22% protein and 35% fat, of which 22% comprised saturated fat. All cheese types contained 1.2% salt and no carbohydrates.

Cheeses were purchased from a local supermarket and stored at 6°C in their original packaging. All experiments were conducted after at least 1 h equilibration at room temperature (approx. 20°C).

### 2.2 Physicochemical Characterization

#### 2.2.1 Color Analysis

The color of the cheese samples was measured (*n* = 6) using a CM‐600d spectrophotometer (Konica Minolta, Japan) with illuminant D65 and reference observer 10° according to the manufacturer's instruction manual (Konica Minolta, n.d.). Cheese color differences were determined by calculating the color difference (ΔE) using Equation (1) (ISO/CIE 2019):

(1) $$\Delta E=\sqrt{{\left(\Delta L\right)}^{2}+{\left(\Delta a\right)}^{2}+{\left(\Delta b\right)}^{2}}$$

where *L*, *a*, and *b* represent coordinates in the CIELAB color space, in the directions of lightness (0 (black) to 100 (white)), green/red (−128 to 127), and blue/yellow (−128 to 127), respectively. As a general guideline, the obtained color difference (Δ*E*) correlates with human visual perception, where a Δ*E* value of approximately 2.3 represents a Just Noticeable Difference (JND) (Sharma and Bala 2017).

To further evaluate the difference in the cheese's yellowness, an index for each product was calculated according to Equation (2) (Francis and Clydesdale 1975):

(2) $$\text{Yellowness Index}=142.83\times \frac{b}{L}$$

where *L* and *b* represent coordinates in the CIELAB color space.

#### 2.2.2 Texture Analysis

Six cubes (approximately 25 × 25 × 25 mm) were cut out from each cheese type, wrapped in plastic film, and maintained at room temperature (20°C–23°C) for 1 h before the analysis.

The texture of the cheeses was analyzed using a CTX texture analyzer (Ametek Brookfield, USA) through a cutting test, following an in‐house method based on the manufacturer's instructions (AMETEK Brookfield Inc 2019). The texture analyzer was equipped with a 5 kg load cell and a 1 mm blade (TA‐SBA‐WB‐1, Ametek Brookfield, USA). The cutting test was performed at a speed of 60 mm/min until 90% of the cheese cubes were sliced. Several parameters, including force at first fracture (*N*; fracturability), maximum force (*N*), and adhesive force (*N*), describing the texture, were obtained from the Texture Pro software (Ametek Brookfield, USA). In gastronomic terms, fracturability, maximum force, and adhesive force can translate to crumbliness/brittleness, hardness, and adhesiveness/stickiness, respectively (Bondu et al. 2022; Khule et al. 2024).

### 2.3 Descriptive Sensory Analysis

Descriptive sensory analysis was performed according to the Quantitative Descriptive Analysis (QDA) method, as outlined by Lawless and Heymann (2010), at the sensory unit of Kristianstad University. The procedure adhered to international standards (including ISO 6658:2017, ISO 8586:2023, ISO 8589:2007, ISO 13299:2016) and involved panel selection, descriptive training, and final sensory assessment.

The selected sensory panel (*n* = 6) was trained during two sessions. A vocabulary of descriptive attributes was generated during the first session, which the panelists were trained to use during the second session. A total of 21 attributes were selected, including five describing the odor, four each for appearance, taste, flavor, and texture (Table 1).

**TABLE 1:** Descriptive sensory attributes of hard cheese, including their definitions, developed by the sensory panel (*n* = 6). The attributes were translated from Swedish to English by the authors of the paper.

	Attribute	Definition
Odor (O)	Apricot	Apricot marmalade
	Odor intensity	Total odor intensity of the sample
	Lactic acid	Crème fraîche
	Nuttiness	Toasted bread or browned butter (beurre noisette)
	Butter	Butter at room temperature
Appearance (A)	Glossiness	Luster of the sample
	White spots	Occurrence of white spots/crystals
	Graininess	Granularity of the sample
	Holes	Size from small to large
Taste (T)	Saltiness	Basic taste
	Sweetness	Basic taste
	Bitterness	Basic taste
	Umami	Basic taste
Flavor (F)	Whey butter	Caramel tones
	Sour milk	Fermented milk (creme fraiche/sour cream)
	Nuttiness	Toasted bread or browned butter (beurre noisette)
	Stale	Feet sweat, bacteria
Texture (Tx)	Chewing resistance	Toughness of sample
	Crumbliness	Tendency to crumble
	Crystals	Crispy crystals/particles
	Greasy mouthfeel	Adhesive in the mouth

In the third session, evaluation of the cheeses was carried out in triplicate using an intensity scale from 0 to 100. The samples, containing 10–15 g of cheese, were presented in plastic cups anonymized with three‐digit codes. Between each sample, panelists were encouraged to rinse their mouths with cold or hot water, and/or neutral crackers.

### 2.4 Affective Sensory Analysis

The consumer study, as described in Lawless and Heymann (2010), was conducted to evaluate consumers' consumption frequency, favorites, and hedonic liking of the studied cheeses. The cheeses were cut into 5.0 ± 0.5 g pieces and presented in plastic cups labeled with randomized three‐digit codes. Samples were prepared in the morning of the test day and kept at room temperature for at least 1 h to allow for temperature equilibration.

The study was carried out during an international conference held at Kristianstad University in the summer of 2024. The inclusion criterion for participation in the test was a minimum age of 18 years. Participants completed an online questionnaire in English, designed using the EyeQuestion platform (version 3.9.7, the Netherlands). In addition to gender and nationality, participants provided background information on their consumption frequency and perceived favorite among the cheeses studied. The consumer group (*n* = 50) comprised 28 women, 21 men, and one person of non‐binary gender. The consumers' age span ranged from 21 to 88 years, with an average age of 45.5 years (median 50 years). Approximately two‐thirds (32 participants) were Swedish, while the remaining one‐third (18 participants) identified as non‐Swedish.

Following the demographic questions, participants rated their liking of the cheese samples using a linear 9‐point hedonic scale. The scale ranged from 1: dislike extremely, 2: dislike very much, 3: dislike moderately, 4: dislike slightly, 5: neither dislike nor like (center point), 6: like slightly, 7: like moderately, 8: like very much, to 9: like extremely. Each sample was evaluated based on appearance, texture, taste, and overall liking.

Participants were encouraged to rinse their palate with water and/or a neutral cracker and wait at least 30 s between evaluations of different samples.

### 2.5 Statistical Analysis

Descriptive statistics were applied to all obtained data in Microsoft Excel (version 2412, USA) and reported as mean values with standard deviation. To determine if any significant (*p* < 0.05) differences occurred between the products, two‐way analysis of variance (ANOVA) followed by Tukey's post hoc test was performed for the descriptive sensory data with panelists and products as fixed factors, and the Friedman's test followed by Durbin‐Conover *post hoc* test was performed for the consumer liking data. These calculations were performed in jamovi (version 2.3.28, Australia) and/or EyeQuestion (version 6.1.2.6, the Netherlands). Friedman's test is a non‐parametric test commonly used when the data violate the assumptions of normality and/or homoscedasticity. Pearson's correlation analysis of average values was conducted to evaluate potential relationships between results from the instrumental color and texture analysis, descriptive sensory study, and consumer liking study. Coefficients larger than 0.7 or smaller than −0.7 (*r* ≥ |0.7|) were considered indicative of strong relationships between two variables. Principal Component Analysis (PCA) was performed in EyeOpenR (version 6.1.2.6, the Netherlands).

## 3 Results

### 3.1 Physicochemical Characterization

#### 3.1.1 Instrumental Color Analysis

**FIGURE 1:**
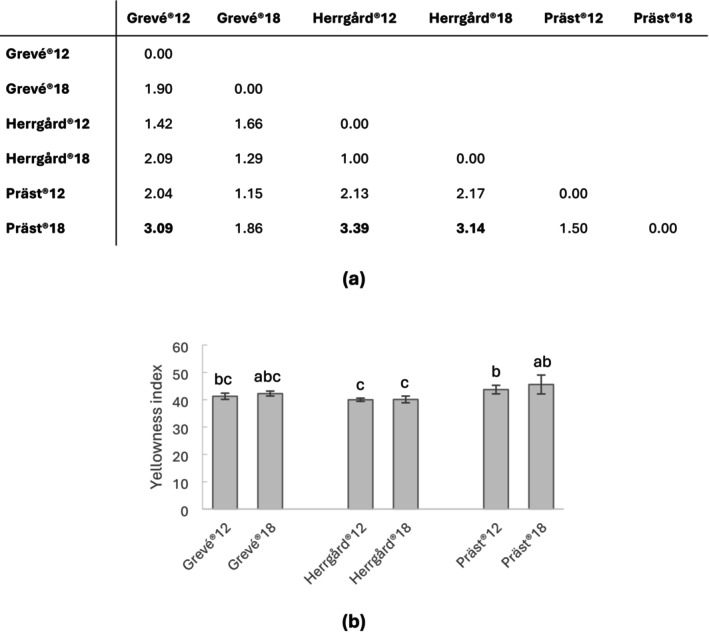
Spectrophotometric color analysis of cheese samples (*n* = 6). (a) Color difference (Δ*E*) between the cheeses. Numbers in bold indicate perceptible differences according to the just noticeable difference (JND) threshold. (b) Yellowness index of the cheese samples. Significant differences (*p* < 0.05; Tukey's) are indicated with letters.

The lowest color difference was observed between Herrgård12 and Herrgård18 (Figure 1a). These two cheese types also proved to have the lowest yellowness index (Figure 1b). The color difference between Präst18 and Grevé12, as well as both maturation levels of Herrgård cheese, was observed at a perceptible level. This difference was also reflected in the yellowness index, which indicated higher yellowness in Präst compared to Herrgård and Grevé.

#### 3.1.2 Instrumental Texture Profiles

**FIGURE 2:**
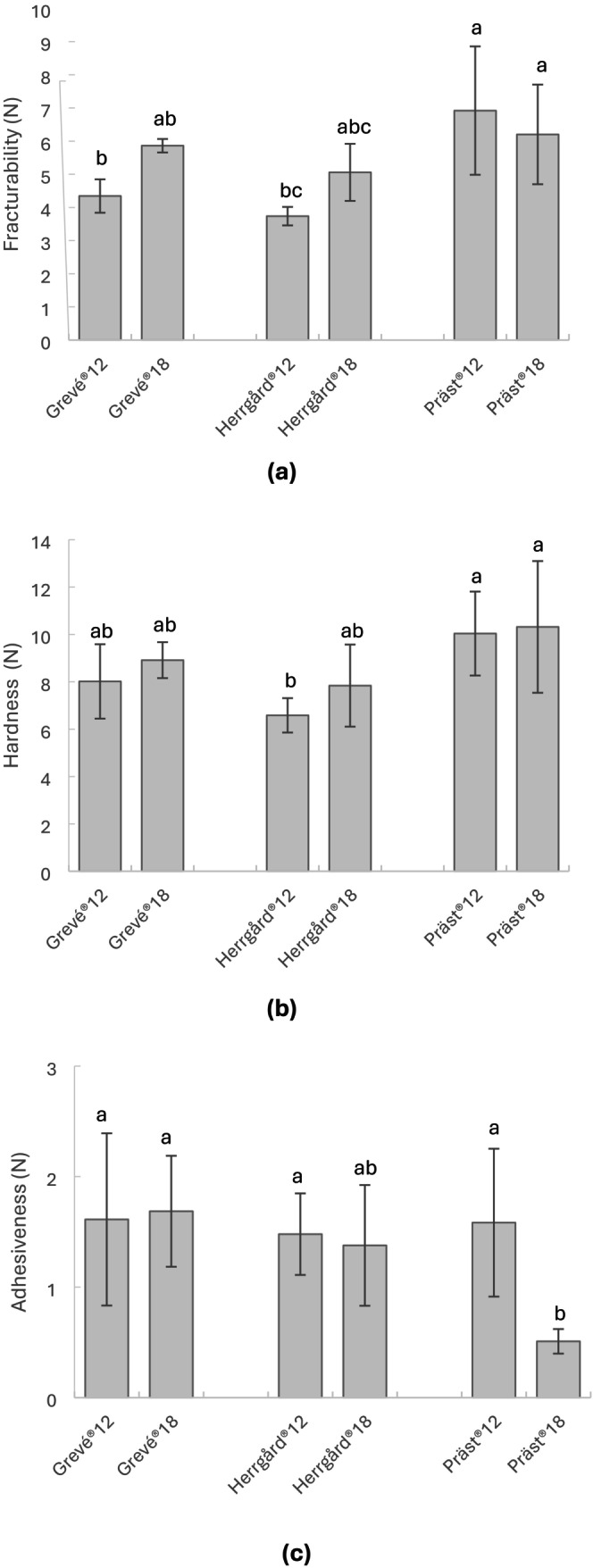
Instrumental texture analysis of cheese samples (*n* = 6), including (a) first fracture (fracturability), (b) maximum force (hardness), and (c) adhesive force (adhesiveness). Values are given as means ± standard deviation, and significant differences (*p* < 0.05; Tukey's) between samples are indicated with letters.

The crumbliness (fracturability; Figure 2a) was generally highest in both Präst cheeses, as well as in the more mature versions of Grevé and Herrgård. Hence, crumbliness tended to increase with cheese maturation, but not at a significant level. Both versions of Präst also proved to be the hardest cheeses (Figure 2b), differing significantly (*p* < 0.05) from Herrgård12, but not from the other cheese samples. In contrast to crumbliness and hardness, Präst18 exhibited significantly (*p* < 0.05) less adhesiveness (Figure 2c), while the other cheese samples appeared similar.

The correlation analysis (Table S2) revealed that hardness and crumbliness (from the sensory panel) were positively associated with fat content (0.850 and 0.894; *p* = 0.032 and *p* = 0.016) and negatively associated with protein content (−0.850 and −0.894; *p* = 0.032 and *p* = 0.016). Fracturability (instrumental crumbliness) and hardness related positively (0.935; *p* = 0.006). Greasy mouthfeel and adhesiveness (instrumental stickiness) showed no significant association with fat content; neither did chewing resistance with hardness, nor crumbliness with fracturability.

### 3.2 Sensory Profiles

The descriptive sensory test generated 21 descriptive attributes, which together create the sensory profiles of the cheeses (Table 2). Among these attributes, five described the odor, and four each described visual appearance, taste, flavor, and texture. Out of the total 21 descriptive attributes, 16 differed significantly (*p* < 0.05) between the studied cheese samples. Sample discrimination was primarily driven by differences in texture and appearance. Chewing resistance was highest in Präst18 and Herrgård12, and lowest in Grevé18 and Herrgård18. While instrumental testing showed Präst cheeses were the hardest, chewing resistance did not correlate with hardness. Both variants of Präst were crumblier than the other cheeses.

**TABLE 2:** Sensory profiles of Grevé, Herrgård, and Präst cheese matured for 12 and 18 months. The heat map illustrates the increased intensity of each attribute as a descriptor for the respective cheese, ranging from red to green. Results are given as mean values with standard deviation. Attributes significantly differing (*p* < 0.05, Tukey's) between samples are highlighted in bold, and these differences are indicated with letters (a–d).

Attributes	Overall	Grevé12	Grevé18	Herrgård12	Herrgård18	Präst12	Präst18	*p*
**O—Apricot**	26.1 ± 16.3	31.4 ± 18.6 **a**	28.4 ± 18.8 **ab**	28.8 ± 12.8 **ab**	25.8 ± 15.8 **ab**	22.5 ± 15.3 **ab**	20.6 ± 15.3 **b**	**0.025**
**O—Odor intensity**	45.3 ± 16.0	49.2 ± 17.7 **ab**	50.1 ± 17.7 **a**	42.8 ± 15.1 **b**	41.0 ± 15.3 **bc**	39.9 ± 11.7 **bc**	45.4 ± 14.9 **abc**	**< 0.001**
**O—Lactic acid**	36.1 ± 10.4	35.0 ± 10.9	38.5 ± 10.5	38.4 ± 10.1	38.0 ± 9.4	37.8 ± 8.2	32.0 ± 9.5	**0.046**
**O—Nuttiness**	42.8 ± 15.1	38.0 ± 14.1 **b**	40.9 ± 16.4 **ab**	44.4 ± 15.6 **ab**	41.0 ± 14.7 **ab**	45.3 ± 18.3 **ab**	47.3 ± 13.9 **a**	**0.012**
O—Butter	42.1 ± 15.9	38.1 ± 12.5	41.3 ± 16.4	46.3 ± 15.7	44.5 ± 16.4	46.6 ± 17.7	42.8 ± 17.9	0.187
**A—Glossiness**	38.5 ± 18.9	31.1 ± 14.0 **bc**	31.5 ± 13.7 **bc**	24.7 ± 11.1 **c**	36.1 ± 20.5 **b**	36.2 ± 17.4 **b**	54.0 ± 16.7 **a**	**< 0.001**
**A—Graininess**	24.4 ± 20.0	14.6 ± 10.8 **b**	13.3 ± 9.0 **b**	15.2 ± 8.7 **b**	14.3 ± 11.7 **b**	53.6 ± 16.9 **a**	50.1 ± 15.3 **a**	**< 0.001**
**A—White spots**	14.8 ± 11.3	8.0 ± 4.9 **c**	8.8 ± 7.9 **c**	10.4 ± 5.8 **bc**	16.2 ± 10.7 **b**	12.1 ± 9.0 **bc**	22.8 ± 9.3 **a**	**< 0.001**
**A—Holes**	27.2 ± 17.2	44.6 ± 18.2 **a**	26.7 ± 21.2 **b**	25.4 ± 18.1 **b**	17.2 ± 13.5 **b**	20.6 ± 6.0 **b**	22.9 ± 8.0 **b**	**< 0.001**
**T—Saltiness**	39.4 ± 10.6	34.4 ± 12.4 **bc**	36.1 ± 8.9 **bc**	37.4 ± 11.5 **b**	38.6 ± 12.5 **b**	43.0 ± 6.2 **ab**	45.0 ± 9.5 **a**	**< 0.001**
**T—Sweetness**	25.0 ± 8.5	27.2 ± 7.1 **a**	27.1 ± 6.5 **a**	21.6 ± 7.0 **b**	22.5 ± 8.1 **b**	24.2 ± 8.3 **ab**	22.7 ± 8.0 **b**	**< 0.001**
T—Bitterness	32.4 ± 12.0	31.9 ± 12.8	31.6 ± 11.2	33.4 ± 11.1	32.8 ± 10.4	30.3 ± 14.1	32.5 ± 14.2	0.714
T—Umami	42.0 ± 8.6	40.6 ± 9.7	42.6 ± 8.4	42.1 ± 12.6	42.5 ± 8.1	42.6 ± 5.6	43.2 ± 6.4	0.897
**F—Whey butter**	24.4 ± 15.5	24.1 ± 12.7 **ab**	25.4 ± 15.7 **ab**	21.1 ± 12.0 **b**	29.4 ± 16.6 **a**	25.6 ± 12.6 **ab**	21.6 ± 17.5 **b**	**0.025**
F—Sour milk	39.6 ± 10.5	40.3 ± 11.3	39.7 ± 9.6	41.0 ± 11.0	38.9 ± 11.3	41.3 ± 9.4	39.9 ± 9.4	0.928
F—Nuttiness	46.0 ± 13.8	45.4 ± 13.1	47.3 ± 12.2	45.1 ± 14.1	43.1 ± 12.9	48.2 ± 15.6	46.0 ± 13.7	0.293
**F—Stale**	29.3 ± 19.9	36.2 ± 25.4 **a**	36.5 ± 22.6 **a**	23.7 ± 16.6 **b**	22.5 ± 13.2 **b**	21.8 ± 13.8 **b**	24.8 ± 16.2 **b**	**< 0.001**
**Tx—Chewing resistance**	30.9 ± 15.5	28.0 ± 15.7 **b**	21.8 ± 9.9 **b**	38.2 ± 14.7 **a**	26.3 ± 16.0 **b**	32.6 ± 14.3 **b**	38.1 ± 19.3 **a**	**< 0.001**
**Tx—Crumbliness**	29.6 ± 14.8	26.1 ± 11.5 **b**	18.4 ± 8.0 **bc**	29.4 ± 14.3 **b**	24.5 ± 11.4 **bc**	37.6 ± 12.1 **ab**	45.5 ± 16.7 **a**	**< 0.001**
**Tx—Crystals**	13.5 ± 9.9	7.3 ± 3.7 **c**	8.2 ± 6.9 **c**	8.3 ± 6.6 **c**	16.3 ± 8.8 **b**	9.6 ± 7.0 **bc**	18.6 ± 9.9 **ab**	**< 0.001**
**Tx—Greasy mouthfeel**	53.6 ± 15.5	49.5 ± 15.5	55.8 ± 14.7	54.6 ± 14.6	55.8 ± 14.6	50.8 ± 15.2	48.0 ± 16.6	**0.024**

**FIGURE 3:**
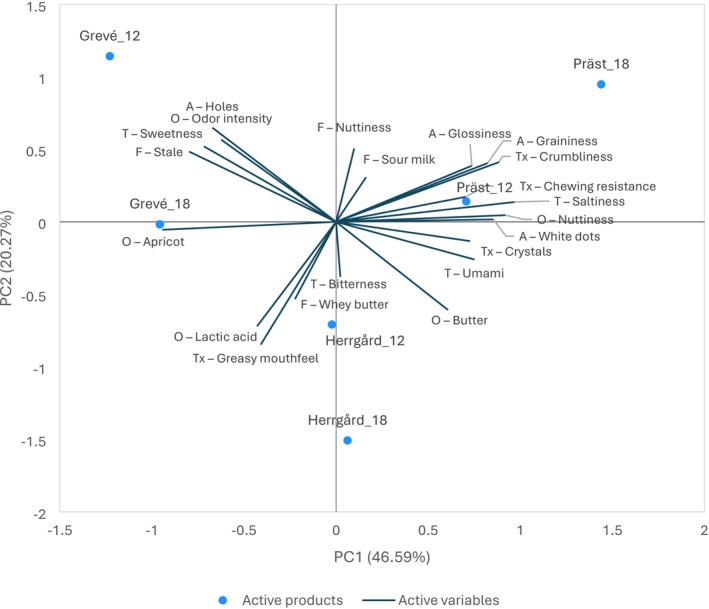
PCA biplot (combining loading plot and score plot) showing the relationship between cheese products and attributes with significant differences among the cheeses (as per Table 2).

The heat map in Table 2 illustrates the intensity of each attribute as a descriptor for the respective cheese, increasing from red to green. Overall, greasy mouthfeel, nuttiness, odor intensity, butter odor, and umami received the highest intensity scores. The relation between samples and their attributes is further illustrated in a PCA biplot (Figure 3). The PCA plot suggests that the sensory profiles are more influenced by cheese type than maturation time.

### 3.3 Consumer Liking

**FIGURE 4:**
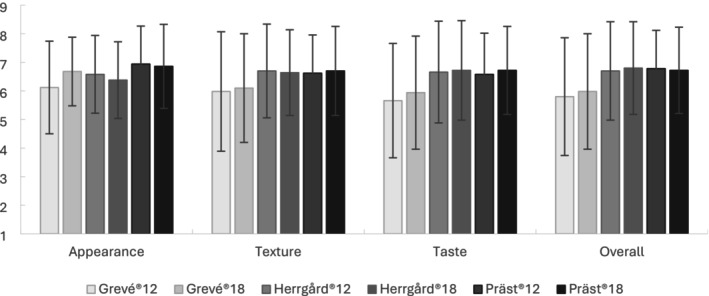
Comparison of the consumers' scores for appearance, texture, taste, and overall liking towards samples of Grevé, Herrgård, and Präst. Scores are presented as mean values with error bars representing standard deviation. See Table S1a–d for significant differences (*p* < 0.05, Durbin‐Conover) between samples.

Overall liking, as evaluated by the hedonic test, indicated that the majority of the consumer group liked the different cheeses presented in the study (Figure 4). Liking scores ranged between “like slightly” (6) to “like very much” (8). Specifically, appearance scores ranged from 6.1 to 6.9, texture from 6.0 to 6.7, taste from 5.7 to 6.7, and overall liking from 5.8 to 6.8, depending on the cheese type. The percentages of participants who gave positive scores (> 5) were 60.0%–84.0% for appearance, 66.0%–84.0% for texture, 58.0%–88.0% for taste, and 62.0%–86.0% for overall liking across all cheeses. Considering all evaluated attributes, Präst18 emerged as the most liked by the consumers, while Grevé12 was the least appreciated. These obtained liking scores agreed with the participants' self‐reported consumption frequencies and favorite cheeses, where Präst was best liked (26%), followed by Herrgård (22%), and lastly Grevé (14%).

The familiarity of the cheese types was, as expected, higher among the Swedes than the non‐Swedes (Table 3). A majority of the participants reported a consumption frequency of the cheeses “less than once a month.” Herrgård was the most frequently consumed cheese among the participants on a daily basis, with 8.0% of participants reporting daily intake. In contrast, Präst was the most consumed cheese on a weekly basis or more, reported by 16.0% of the study population. Overall, a larger variation between cheese types (Grevé, Herrgård, and Präst) was observed than between ripening times (12 and 18 months).

**TABLE 3:** Consumption frequency of the studied cheeses among the participants in the consumer test (*n* = 50, whereof 32 people were Swedish, and 18 non‐Swedish), reported as percent of the whole study population (All), percent of Swedes (S), and percent of non‐Swedes (NS).

Consumption frequency	Grevé			Herrgård			Präst		
	All	S	NS	All	S	NS	All	S	NS
Everyday	2.0	3.1	0.0	8.0	12.5	0.0	0.0	0.0	0.0
Once a week or more	8.0	12.5	0.0	2.0	3.1	0.0	16.0	21.9	5.6
Once a month or more	10.0	12.5	5.6	28.0	37.5	11.1	28.0	43.8	0.0
Less than once a month	52.0	71.9	16.7	52.0	46.9	0.0	52.0	34.4	11.1
Do not know this cheese	28.0	0.0	77.8	30.0	0.0	83.3	30.0	0.0	83.3

Overall liking appeared to be influenced by the taste (0.987; *p* < 0.001) and texture (0.984; *p* < 0.001) of the cheeses, while appearance (0.546; *p* = 0.263) did not generally have any significant impact (Table S2a–c). Overall liking was negatively influenced by the size of holes (−0.846; *p* = 0.034), odor intensity (−0.905; *p* = 0.013) which appeared affected by stale aromas, sweetness (−0.912; *p* = 0.011) and stale flavor (−0.985; *p* < 0.001), and positively by butter odor (0.883; *p* = 0.020). In addition, the results indicated trends of positive influence of nuttiness (0.753; *p* = 0.084) and saltiness (0.730; *p* = 0.100) on the overall liking.

## 4 Discussion

Perceived sensory quality is an important factor for consumer liking (Ojeda et al. 2021). In this study, taste and texture attributes proved more important for overall liking than appearance. In general, butter odor, and partly nuttiness and saltiness, proved to be hallmarks for liked cheeses, while hole size, odor intensity, sweetness, and stale flavor had negative connotations. According to Yates and Drake (2007), overall liking increases with fat content to a certain level. This is in line with the present study, where Präst cheese was best liked by the consumers and also has the highest fat content. Both fat and protein content appeared to influence the crumbliness of the cheeses. However, to verify these interactions, a more detailed analysis of the composition and physicochemical structures is required.

Most of the attributes developed in the training sessions are recurring in several other studies to describe hard cheeses (Caspia et al. 2006; Castada et al. 2019; Muir et al. 1995). The hole size is a result of the manufacturing and maturation processes. Präst cheese is stirred while the whey is drained, resulting in small air bubbles. Contrarily, the curd of Grevé and Herrgård is pressed to drain the whey, resulting in no air bubbles. Instead, the holes develop later during maturation due to the production of carbon dioxide by bacteria (Nilsson Blom and Weréen 2002; Zheng et al. 2021). Hence, and in line with Fibri et al. (2022), manufacturing and maturation processes should be refined for cheeses with enhanced consumer acceptance. It should also be noted that although the application of modern biotechnology, such as fermentation techniques, has transformed the ancient methods of producing traditional fermented foods, these innovations have also been employed to extend food shelf‐life (Fibri et al. 2022).

The obtained results showed a larger variation between cheese types (Grevé, Herrgård, and Präst) than between ripening times (12 and 18 months). A noticeable difference between cheeses of various ripening times is expected since new odor and flavor compounds develop during maturation (Caspia et al. 2006; D'Incecco et al. 2020). These new odors and flavors are the consequence of proteolysis and lipolysis, which degrade casein into peptides and free amino acids (D'Incecco et al. 2020). Hence, a more pronounced effect of the maturation process might have been observed if cheeses with a larger maturation gap had been included. In addition, the number of white spots increases with maturation (D'Incecco et al. 2020) as a result of slow water migration within the cheese matrix, which leads to the compartmentalization of hydrophobic free fatty acids. This phenomenon was mainly observed in Präst18. Meanwhile, the yellowness index tended to be similar between cheeses matured for 12 and 18 months. While D'Incecco et al. (2020) suggest that the yellowness index does not relate to cheese age, Rohm and Jaros (1996) show that the yellowness index of hard cheese increases during the first 10 to 13 weeks of ripening. The yellowness level is influenced by several parameters, for example the breed and diet of the cows (McGuinness et al. 2024), deduced from the variation of β‐carotene and carotenoid levels in their diet (D'Incecco et al. 2020). The yellowness index did not substantially influence the liking in this study.

The prospects for Swedish traditional cheeses indicate a potential for increased consumption, based on the study group's frequency report. Understanding the characteristics of traditional cheeses and their consumer acceptance can facilitate the usability and development of local products. It also appears that these cheeses are not widely recognized internationally (although based on a few consumers), leaving a gap for expansion. Traditional foods bring cultural value, which Keskin and Dağ (2020) demonstrate with Turkish regional cheeses. They proposed that protecting traditional cheeses and their distinctive perceptual qualities can aid in preserving cultural and regional heritage, while also promoting gastronomic tourism. Guerrero et al. (2010) have observed that people from Southern European regions associate traditional foods with heritage, culture, or history, while inhabitants of Central and Nordic European regions focus on practical issues such as convenience, health, or appropriateness.

Liking of food has been shown to relate to the familiarity of specific food products (Frez‐Muñoz et al. 2024; Ojeda et al. 2021). In this study, one‐third of the participating consumers came from mainly other European countries and had little to no experience with Swedish traditional cheeses. Despite this, a majority of the consumers liked all the cheeses, indicating that various factors influence liking. Yet, both familiarity and liking of the cheeses were generally higher among Swedes. Ojeda et al. (2021) report that local cheeses are perceived to have higher sensory quality than non‐local cheeses. In line with this, quality labels such as PDO (Protected Designation of Origin) and PGI (Protected Geographical Indication) (European Commission 2025) could potentially increase consumer interest in traditional cheese and enhance its consumption frequency (Braghieri et al. 2014). Further, for maintaining the right quality and character of the food, Fibri et al. (2022) claim that labeling is needed for traditional foods.

It must be further noted that the production of animal‐based cheese contributes significantly to the negative environmental impact of our food (Finnegan et al. 2018; Our World in Data 2024; Poore and Nemecek 2018; Üçtuğ 2019). To make one kilogram of hard cheese, ten kilograms of milk are required (Nilsson Blom and Weréen 2002). Current data suggest that plant‐based cheese production has lower carbon emissions and is better for animal welfare (Carlsson Kanyama et al. 2021; Grossmann and McClements 2021). Hence, knowledge about sensory profiles of animal‐based cheese can serve as a foundation for the development of improved plant‐based analogues (Alehosseini et al. 2025; Genet et al. 2023) with a lower carbon footprint and reduced exploitation of animals.

A study by Ojeda et al. (2021) showed that the perceived quality and liking of traditional cheeses were higher in comparison to non‐traditional cheeses. Thus, new cheese innovations, including plant‐based analogues, may need to develop in the direction of familiarity and tradition (Guiné et al. 2021). Yet, the modification and modernization of traditional foods are also strategic approaches to meeting evolving consumer needs (Fibri et al. 2022). Ethical structures and gastronomic traditions are important in most cultures (Rajan 2023) and have also been shown to attract tourists (Frez‐Muñoz et al. 2024; Grubor et al. 2022). Thus, it seems that traditional foods, such as cheese, have an important role to play in maintaining cultural identity.

Although this study provides valuable insights into the sensory profiles of three types of traditional hard cheese and consumer acceptance towards these, it is important to recognize the limited number of participants recruited. A trained panel should be sufficiently large to ensure that the most critical attributes are accurately evaluated (Silva et al. 2014), which is typically 8 to 12 judges (Lawless and Heymann 2010). Studies may, however, employ panels of varying sizes, with six to 20 participants normally being considered acceptable (Silva et al. 2014). It should be noted that the expert panel employed at Kristianstad University undergoes selection tests before recruitment, yearly checks, and specific training prior to assessment to integrate quality into the sensory studies. Still, studies relying on human assessors can introduce variability in results, as in Table 2. Similarly, depending on the power calculations and test setup, a consumer panel should typically comprise 50 to 300 participants (Hough et al. 2006; Meilgaard et al. 1999).

## 5 Conclusion

Food producers aim to develop new products aligning with contemporary needs and trends. However, for traditional foods, maintenance of characteristics is important. Consumers generally liked all three traditional cheeses, Grevé, Herrgård, and Präst, with a majority of participants giving positive scores. The findings indicated that taste and texture were the primary drivers of overall liking, while appearance played a lesser role. Butter odor, and partly nuttiness and saltiness, proved to be hallmarks for liked cheeses, while hole size, odor intensity, sweetness, and stale flavor had negative connotations. Präst and Herrgård were overall liked better than Grevé, reflecting current consumption patterns. Differences in liking may be explained by variations in physicochemical properties, with Präst being more yellow, crumbly, and hard, and Grevé being stickier. Labeling of these products for their originality could be of importance for increased consumer interest. The understanding of traditional cheeses' characteristics and their consumer acceptance can facilitate the usability and development of new and local products to be further developed in the directions of traditions, familiarity, and sustainability.

## Author Contributions

**Julie Hirtz:** formal analysis (equal), investigation (equal), visualization (equal), writing – original draft (equal), writing – review and editing (equal). **Kalyani Douraquia:** formal analysis (equal), investigation (equal), writing – original draft (equal), writing – review and editing (equal). **Madeleine Jönsson:** formal analysis (equal), visualization (equal), writing – original draft (equal), writing – review and editing (equal). **Karin Wendin:** conceptualization (equal), funding acquisition (equal), methodology (equal), project administration (equal), resources (equal), supervision (equal), writing – review and editing (equal).

## Ethics Statement

The present study, which evaluates sensory perceptions of food, does not encompass sensitive personal data as defined by the Swedish Data Protection Act (2018:218) and Ordinance (2018:219). Participation was voluntary, and all participants gave their informed consent to participate before the study, after being informed about the test and the contents of the assessed products, including allergens. Participation in the consumer test was anonymous, and no information from the test can be traced to or used to identify any individual participant, in accordance with the General Data Protection Regulation (GDPR).

## Conflicts of Interest

The authors declare no conflicts of interest.

## Supporting information


**Table S1:** (a) Pairwise comparisons (Durbin‐Conover) of appearance. (b) Pairwise comparisons (Durbin‐Conover) of texture. (c) Pairwise comparisons (Durbin‐Conover) of taste. (d) Pairwise comparisons (Durbin‐Conover) of overall liking.
**Table S2:** (a) Pearson's correlation of appearance related measurements. (b) Pearson's correlation of texture related measurements. (c) Pearson's correlation of measurements related to odor, taste and flavor.

## Data Availability

The data that support the findings of this study are available from the corresponding author upon reasonable request.

## References

[fsn370913-bib-0001] Alehosseini, E. , P. L. H. McSweeney , and S. Miao . 2025. “Recent Updates on Plant Protein‐Based Dairy Cheese Alternatives: Outlook and Challenges.” Critical Reviews in Food Science and Nutrition: 1–15. 10.1080/10408398.2025.2452356. 39819182

[fsn370913-bib-0002] AMETEK Brookfield Inc . 2019. “CTX Texture Analyzer: Operating Instructions.” https://www.brookfieldengineering.com/‐/media/ametekbrookfield/product‐manuals/ctx‐texture‐analyzer‐operations‐manual‐m19‐373.pdf?la=en&revision=1ea2727c‐e3a3‐475f‐8626‐0c3ece045deb&hash=9467EC92B4D63CF314CE8E4D080CCA7A.

[fsn370913-bib-0003] Bondu, C. , C. Salles , M. Weber , E. Guichard , and M. Visalli . 2022. “Construction of a Generic and Evolutive Wheel and Lexicon of Food Textures.” Food 11, no. 19: 3097. 10.3390/foods11193097. PMC9562153 36230172

[fsn370913-bib-0004] Braghieri, A. , A. Girolami , A. M. Riviezzi , N. Piazzolla , and F. Napolitano . 2014. “Liking of Traditional Cheese and Consumer Willingness to Pay.” Italian Journal of Animal Science 13, no. 1: 3029. 10.4081/ijas.2014.3029.

[fsn370913-bib-0005] Carlsson Kanyama, A. , B. Hedin , and C. Katzeff . 2021. “Differences in Environmental Impact Between Plant‐Based Alternatives to Dairy and Dairy Products: A Systematic Literature Review.” Sustainability 13, no. 22: 12599. https://www.mdpi.com/2071‐1050/13/22/12599.

[fsn370913-bib-0006] Caspia, E. , P. Coggins , M. Schilling , Y. Yoon , and C. White . 2006. “The Relationship Between Consumer Acceptability and Descriptive Sensory Attributes in Cheddar Cheese.” Journal of Sensory Studies 21, no. 1: 112–127. 10.1111/j.1745-459X.2006.00054.x.

[fsn370913-bib-0007] Castada, H. Z. , K. Hanas , and S. A. Barringer . 2019. “Swiss Cheese Flavor Variability Based on Correlations of Volatile Flavor Compounds, Descriptive Sensory Attributes, and Consumer Preference.” Food 8, no. 2: 78. https://www.mdpi.com/2304‐8158/8/2/78. 10.3390/foods8020078 PMC6406939 30791411

[fsn370913-bib-0008] D'Incecco, P. , S. Limbo , F. Faoro , et al. 2016. “New Insight on Crystal and Spot Development in Hard and Extra‐Hard Cheeses: Association of Spots With Incomplete Aggregation of Curd Granules.” Journal of Dairy Science 99, no. 8: 6144–6156. 10.3168/jds.2016-11050. 27236764

[fsn370913-bib-0009] D'Incecco, P. , S. Limbo , J. Hogenboom , V. Rosi , S. Gobbi , and L. Pellegrino . 2020. “Impact of Extending Hard‐Cheese Ripening: A Multiparameter Characterization of Parmigiano Reggiano Cheese Ripened up to 50 Months.” Food 9, no. 3: 268. 10.3390/foods9030268. PMC7143483 32131400

[fsn370913-bib-0010] European Commission . 2025. “Geographical Indications Food and Drink.” https://agriculture.ec.europa.eu/farming/geographical‐indications‐and‐quality‐schemes/geographical‐indications‐food‐and‐drink_en.

[fsn370913-bib-0011] Fibri, D. L. N. , S. Ayouaz , R. F. Utami , and D. R. A. Muhammad . 2022. “Current Situation and Future Direction of Traditional Foods: A Perspective Review.” Canrea Journal: Food Technology, Nutritions, and Culinary Journal 5: 112–126. 10.20956/canrea.v5i1.633.

[fsn370913-bib-0012] Finnegan, W. , M. Yan , N. M. Holden , and J. Goggins . 2018. “A Review of Environmental Life Cycle Assessment Studies Examining Cheese Production.” International Journal of Life Cycle Assessment 23, no. 9: 1773–1787. 10.1007/s11367-017-1407-7.

[fsn370913-bib-0013] Foegeding, E. A. , and M. A. Drake . 2007. “Invited Review: Sensory and Mechanical Properties of Cheese Texture.” Journal of Dairy Science 90, no. 4: 1611–1624. 10.3168/jds.2006-703. 17369201

[fsn370913-bib-0014] Francis, F. J. , and F. M. Clydesdale . 1975. Food Colorimetry: Theory and Applications. AVI Publishing Co. Inc.

[fsn370913-bib-0015] Frez‐Muñoz, L. , V. Fogliano , and B. L. P. A. Steenbekkers . 2024. “Consumers' Familiarity Level Shapes Motives and Contexts for Preparing and Consuming Dishes.” Journal of Food Science 89, no. 10: 6677–6693. 10.1111/1750-3841.17299. 39215518

[fsn370913-bib-0016] Genet, B. M. L. , G. E. Sedó Molina , A. P. Wätjen , et al. 2023. “Hybrid Cheeses—Supplementation of Cheese With Plant‐Based Ingredients for a Tasty, Nutritious and Sustainable Food Transition.” Fermentation 9, no. 7: 667. https://www.mdpi.com/2311‐5637/9/7/667.

[fsn370913-bib-0017] Grossmann, L. , and D. J. McClements . 2021. “The Science of Plant‐Based Foods: Approaches to Create Nutritious and Sustainable Plant‐Based Cheese Analogs.” Trends in Food Science & Technology 118: 207–229. 10.1016/j.tifs.2021.10.004.

[fsn370913-bib-0018] Grubor, B. , B. Kalenjuk Pivarski , B. Đerčan , et al. 2022. “Traditional and Authentic Food of Ethnic Groups of Vojvodina (Northern Serbia)—Preservation and Potential for Tourism Development.” Sustainability 14, no. 3: 1805. https://www.mdpi.com/2071‐1050/14/3/1805.

[fsn370913-bib-0019] Guerrero, L. , A. Claret , W. Verbeke , et al. 2010. “Perception of Traditional Food Products in Six European Regions Using Free Word Association.” Food Quality and Preference 21, no. 2: 225–233. 10.1016/j.foodqual.2009.06.003.

[fsn370913-bib-0020] Guiné, R. P. F. , S. G. Florença , M. J. Barroca , and O. Anjos . 2021. “The Duality of Innovation and Food Development Versus Purely Traditional Foods.” Trends in Food Science & Technology 109: 16–24. 10.1016/j.tifs.2021.01.010.

[fsn370913-bib-0021] Hough, G. , I. Wakeling , A. Mucci , E. Chambers , I. M. Gallardo , and L. R. Alves . 2006. “Number of Consumers Necessary for Sensory Acceptability Tests.” Food Quality and Preference 17, no. 6: 522–526. 10.1016/j.foodqual.2005.07.002.

[fsn370913-bib-0022] ISO/CIE . 2019. “ISO/CIE 11664‐4: 2019; Colorimetry—Part 4: CIE 1976 L* a* b* Colour Space.”

[fsn370913-bib-0023] Keskin, E. , and T. Dağ . 2020. “Identity of Cheese: A Research on the Cheeses of the Aegean Region in Turkey.” Journal of Ethnic Foods 7, no. 1: 25. 10.1186/s42779-020-00062-4.

[fsn370913-bib-0024] Khule, G. , A. Ranvare , A. Singh , and C. Babu . 2024. “Texture Profile Analysis: A Comprehensive Insight Into Food Texture Evaluation.” Journal of Dynamic Systems, Measurement, and Control: 30–45. 10.71058/jodac.v8i9003.

[fsn370913-bib-0025] Kindstedt, P. 2012. Cheese and Culture: A History of Cheese and Its Place in Western Civilization. Chelsea Green Publishing.

[fsn370913-bib-0026] Konica Minolta . n.d. “Spectrophotometer CM‐700d/600d: Instruction Manual (Japan: Konica Minolta Sensing, Issue).” https://www.konicaminolta.com/instruments/download/instruction_manual/color/pdf/cm‐700d_instruction_eng.pdf.

[fsn370913-bib-0027] Lawless, H. T. , and H. Heymann . 2010. Sensory Evaluation of Food. Food Science Text Series. Springer.

[fsn370913-bib-0028] McGuinness, L. , M. Timlin , J. P. Murphy , et al. 2024. “Impact of Feeding Regimes and Lactation Stage on Sensory Attributes of Cheddar Cheese.” Food Research International 180: 114046. 10.1016/j.foodres.2024.114046. 38395564

[fsn370913-bib-0029] Meilgaard, M. C. , B. T. Carr , and G. V. Civille . 1999. Sensory Evaluation Techniques. CRC Press.

[fsn370913-bib-0030] Muir, D. D. , E. A. Hunter , J. M. Banks , and D. S. Horne . 1995. “Sensory Properties of Hard Cheese: Identification of Key Attributes.” International Dairy Journal 5, no. 2: 157–177. 10.1016/0958-6946(95)92208-L.

[fsn370913-bib-0031] Nilsson Blom, U.‐K. , and P.‐O. Weréen . 2002. “Cheese and Cheese‐Making—With a Special Emphasis on Swedish Cheeses (Bioscience Explained, Issue 2).” https://bioscience‐explained.org/content/cheeseEN.pdf.

[fsn370913-bib-0032] Ojeda, M. , I. Etaio , D. Valentin , et al. 2021. “Effect of Consumers' Origin on Perceived Sensory Quality, Liking and Liking Drivers: A Cross‐Cultural Study on European Cheeses.” Food Quality and Preference 87: 104047. 10.1016/j.foodqual.2020.104047.

[fsn370913-bib-0033] Our World in Data . 2024. “Environmental Impacts of Food Data Explorer.” https://ourworldindata.org/environmental‐impacts‐of‐food#explore‐data‐on‐the‐environmental‐impacts‐of‐food.

[fsn370913-bib-0034] Poore, J. , and T. Nemecek . 2018. “Reducing Food's Environmental Impacts Through Producers and Consumers.” Science 360, no. 6392: 987–992. 10.1126/science.aaq0216. 29853680

[fsn370913-bib-0035] Rajan, A. 2023. “Gastronomic Evolution: A Review of Traditional and Contemporary Food Culture.” International Journal for Multidimensional Research Perspectives 1, no. 2: 62–76.

[fsn370913-bib-0036] Rocillo‐Aquino, Z. , F. Cervantes‐Escoto , J. A. Leos‐Rodríguez , D. Cruz‐Delgado , and A. Espinoza‐Ortega . 2021. “What Is a Traditional Food? Conceptual Evolution From Four Dimensions.” Journal of Ethnic Foods 8, no. 1: 38. 10.1186/s42779-021-00113-4.

[fsn370913-bib-0037] Rohm, H. , and D. Jaros . 1996. “Colour of Hard Cheese.” Zeitschrift für Lebensmittel‐Untersuchung und ‐Forschung 203, no. 3: 241–244. 10.1007/BF01192871.

[fsn370913-bib-0038] Sharma, G. , and R. Bala . 2017. Digital Color Imaging Handbook. CRC Press.

[fsn370913-bib-0039] Silva, R. C. S. N. , V. P. R. Minim , A. N. d. Silva , and L. A. Minim . 2014. “Number of Judges Necessary for Descriptive Sensory Tests.” Food Quality and Preference 31: 22–27. 10.1016/j.foodqual.2013.07.010.

[fsn370913-bib-0040] Svenska Ostklassiker AB . 2020a. “Grevé—En svensk hårdost som är lite söt, en aning nöt.” https://www.svenskaostklassiker.se/grev%C3%A9/.

[fsn370913-bib-0041] Svenska Ostklassiker AB . 2020b. “Herrgård—En svensk hårdost som är mild och folklig.” https://www.svenskaostklassiker.se/herrgaard/.

[fsn370913-bib-0042] Svenska Ostklassiker AB . 2020c. “Präst—En svensk hårdost som är gräddig med fin sälta.” https://www.svenskaostklassiker.se/praest/.

[fsn370913-bib-0043] Svenskmärkning AB . 2024. “Ost för alla smaker—till vardag och fest.” https://fransverige.se/svenska‐ravaror‐all‐varldens‐mat/vilka‐varor‐marks/svenska‐ravaror/ost/.

[fsn370913-bib-0044] Üçtuğ, F. G. 2019. “The Environmental Life Cycle Assessment of Dairy Products.” Food Engineering Reviews 11, no. 2: 104–121. 10.1007/s12393-019-9187-4.

[fsn370913-bib-0045] Yates, M. , and M. Drake . 2007. “Texture Properties of Gouda Cheese.” Journal of Sensory Studies 22, no. 5: 493–506. 10.1111/j.1745-459X.2007.00124.x.

[fsn370913-bib-0046] Zheng, X. , X. Shi , and B. Wang . 2021. “A Review on the General Cheese Processing Technology, Flavor Biochemical Pathways and the Influence of Yeasts in Cheese.” Frontiers in Microbiology 12: 703284. 10.3389/fmicb.2021.703284. 34394049 PMC8358398

