# Supplementary material for: Taste of Tradition: Perceptual Quality Aspects and Future Prospects of Three Swedish Classic Cheeses
Source: Food Sci Nutr. 2025 Sep 4;13(9):e70913. doi: 10.1002/fsn3.70913 (PMC12411249; doi:10.1002/fsn3.70913)

Supplementary Material

**Taste of Tradition: Perceptual Quality Aspects and Future Prospects of Three Swedish Classic Cheeses**

**Repeated Measures ANOVA (Non-parametric)**

| **Table S1a**. Pairwise Comparisons (Durbin-Conover) of appearance. | | | | |
| --- | --- | --- | --- | --- |
|  |  |  | **Statistic** | **p** |
| **A-G12A** | **-** | **A-G18A** | **2.781** | **0.006** |
| **A-G12A** | **-** | **A-H12A** | **2.296** | **0.023** |
| A-G12A | - | A-H18A | 0.970 | 0.333 |
| **A-G12A** | **-** | **A-P12A** | **4.268** | **<.001** |
| **A-G12A** | **-** | **A-P18A** | **4.042** | **<.001** |
| A-G18A | - | A-H12A | 0.485 | 0.628 |
| A-G18A | - | A-H18A | 1.811 | 0.071 |
| A-G18A | - | A-P12A | 1.487 | 0.138 |
| A-G18A | - | A-P18A | 1.261 | 0.208 |
| A-H12A | - | A-H18A | 1.326 | 0.186 |
| **A-H12A** | **-** | **A-P12A** | **1.972** | **0.050** |
| A-H12A | - | A-P18A | 1.746 | 0.082 |
| **A-H18A** | **-** | **A-P12A** | **3.298** | **0.001** |
| **A-H18A** | **-** | **A-P18A** | **3.072** | **0.002** |
| A-P12A | - | A-P18A | 0.226 | 0.821 |

| **Table S1b**. Pairwise Comparisons (Durbin-Conover) of texture. | | | | |
| --- | --- | --- | --- | --- |
|  |  |  | **Statistic** | **p** |
| Tx-G12A | - | Tx-G18A | 0.885 | 0.377 |
| **Tx-G12A** | **-** | **Tx-H12A** | **3.175** | **0.002** |
| **Tx-G12A** | **-** | **Tx-H18A** | **2.992** | **0.003** |
| **Tx-G12A** | **-** | **Tx-P12A** | **2.534** | **0.012** |
| **Tx-G12A** | **-** | **Tx-P18A** | **2.687** | **0.008** |
| **Tx-G18A** | **-** | **Tx-H12A** | **2.290** | **0.023** |
| **Tx-G18A** | **-** | **Tx-H18A** | **2.106** | **0.036** |
| Tx-G18A | - | Tx-P12A | 1.649 | 0.101 |
| Tx-G18A | - | Tx-P18A | 1.801 | 0.073 |
| Tx-H12A | - | Tx-H18A | 0.183 | 0.855 |
| Tx-H12A | - | Tx-P12A | 0.641 | 0.522 |
| Tx-H12A | - | Tx-P18A | 0.488 | 0.626 |
| Tx-H18A | - | Tx-P12A | 0.458 | 0.647 |
| Tx-H18A | - | Tx-P18A | 0.305 | 0.760 |
| Tx-P12A | - | Tx-P18A | 0.153 | 0.879 |

| **Table S1c**. Pairwise Comparisons (Durbin-Conover) of taste. | | | | |
| --- | --- | --- | --- | --- |
|  |  |  | **Statistic** | **p** |
| T-G12A | - | T-G18A | 0.8676 | 0.386 |
| **T-G12A** | **-** | **T-H12A** | **3.0816** | **0.002** |
| **T-G12A** | **-** | **T-H18A** | **3.4406** | **<.001** |
| **T-G12A** | **-** | **T-P12A** | **2.6627** | **0.008** |
| **T-G12A** | **-** | **T-P18A** | **3.4107** | **<.001** |
| **T-G18A** | **-** | **T-H12A** | **2.2139** | **0.028** |
| **T-G18A** | **-** | **T-H18A** | **2.5730** | **0.011** |
| T-G18A | - | T-P12A | 1.7951 | 0.074 |
| **T-G18A** | **-** | **T-P18A** | **2.5430** | **0.012** |
| T-H12A | - | T-H18A | 0.3590 | 0.720 |
| T-H12A | - | T-P12A | 0.4189 | 0.676 |
| T-H12A | - | T-P18A | 0.3291 | 0.742 |
| T-H18A | - | T-P12A | 0.7779 | 0.437 |
| T-H18A | - | T-P18A | 0.0299 | 0.976 |
| T-P12A | - | T-P18A | 0.7480 | 0.455 |

| **Table S1d**. Pairwise Comparisons (Durbin-Conover) of overall liking. | | | | |
| --- | --- | --- | --- | --- |
|  |  |  | **Statistic** | **p** |
| Ov-G12A | - | Ov-G18A | 0.8096 | 0.419 |
| **Ov-G12A** | **-** | **Ov-H12A** | **3.0286** | **0.003** |
| **Ov-G12A** | **-** | **Ov-H18A** | **3.5684** | **<.001** |
| **Ov-G12A** | **-** | **Ov-P12A** | **2.8487** | **0.005** |
| **Ov-G12A** | **-** | **Ov-P18A** | **3.0586** | **0.002** |
| **Ov-G18A** | **-** | **Ov-H12A** | **2.2190** | **0.027** |
| **Ov-G18A** | **-** | **Ov-H18A** | **2.7587** | **0.006** |
| **Ov-G18A** | **-** | **Ov-P12A** | **2.0391** | **0.043** |
| **Ov-G18A** | **-** | **Ov-P18A** | **2.2490** | **0.025** |
| Ov-H12A | - | Ov-H18A | 0.5398 | 0.590 |
| Ov-H12A | - | Ov-P12A | 0.1799 | 0.857 |
| Ov-H12A | - | Ov-P18A | 0.0300 | 0.976 |
| Ov-H18A | - | Ov-P12A | 0.7197 | 0.472 |
| Ov-H18A | - | Ov-P18A | 0.5098 | 0.611 |
| Ov-P12A | - | Ov-P18A | 0.2099 | 0.834 |

**Table S2a.** Pearson’s correlation of appearance related measurements.


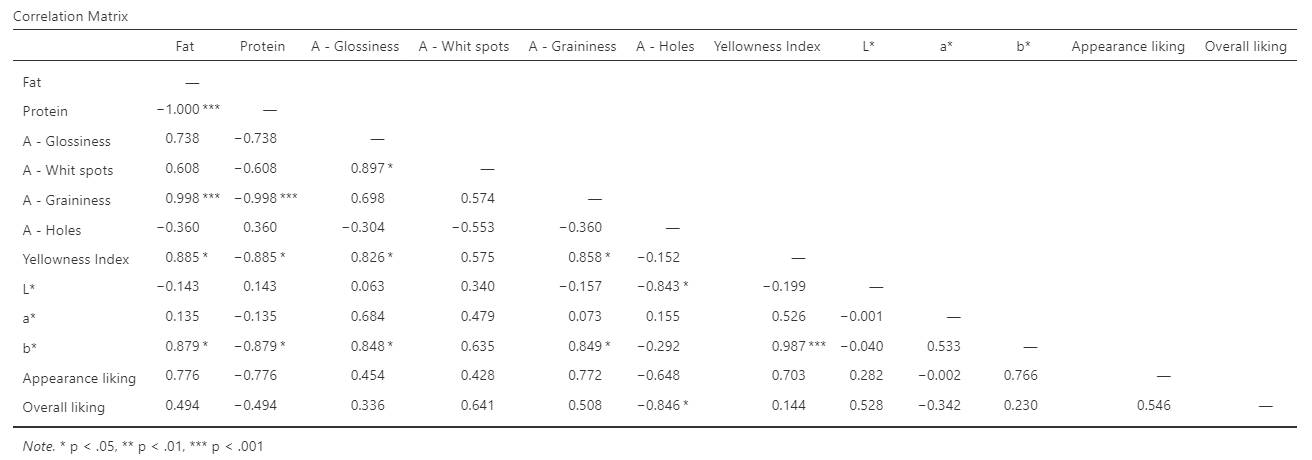


**Table S2b.** Pearson’s correlation of texture related measurements.


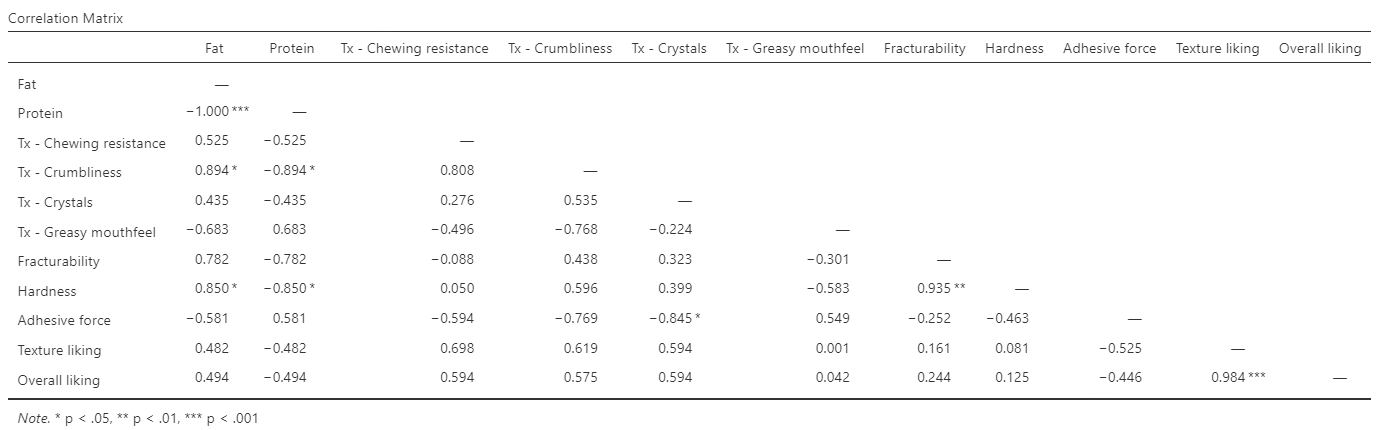


**Table S2c.** Pearson’s correlation of measurements related to odor, taste and flavor.


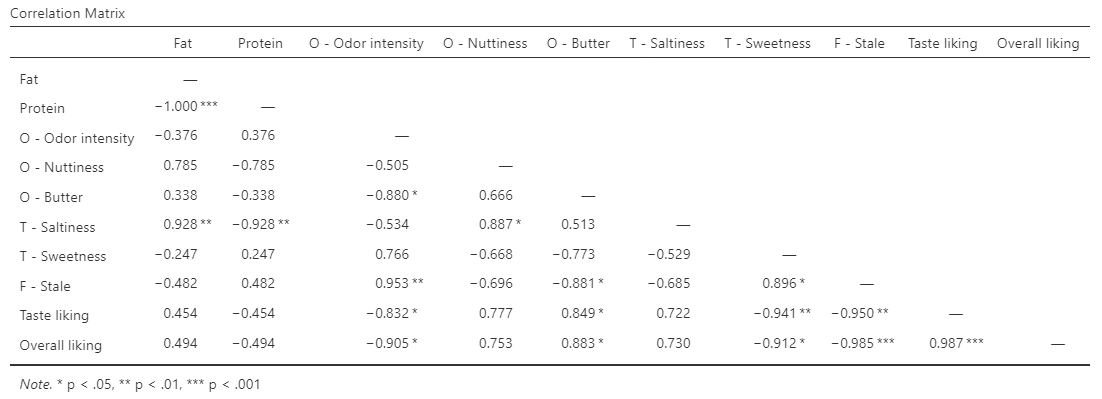

Supplement: Supplementary file 1 — Table S1: (a) Pairwise comparisons (Durbin‐Conover) of appearance. (b) Pairwise comparisons (Durbin‐Conover) of texture. (c) Pairwise comparisons (Durbin‐Conover) of taste. (d) Pairwise comparisons (Durbin‐Conover) of overall liking. Table S2: (a) Pearson's correlation of appearance related measurements. (b) Pearson's correlation of texture related measurements. (c) Pearson's correlation of measurements related to odor, taste and flavor. [file FSN3-13-e70913-s001.docx]
